# Supplementary material for: FERN – a Java framework for stochastic simulation and evaluation of reaction networks
Source: BMC Bioinformatics. 2008 Aug 29;9:356. doi: 10.1186/1471-2105-9-356 (PMC2553347; doi:10.1186/1471-2105-9-356)
Supplement: Additional file 1 — FERN distribution, Version 1.3. This archive contains the FERN source code and binaries as well as documentation and example models in FernML and SBML. [file 1471-2105-9-356-S1.zip › fern/doc/javadoc/fern/simulation/controller/AndController.html]

AndController


---


|  |  |  |
| --- | --- | --- |
| **Overview** \| **Package** \| **Class** \| **Use** \| **Tree** \| **Deprecated** \| **Index** \| **Help** | |  |
| PREV CLASS   **NEXT CLASS** | **FRAMES**    **NO FRAMES**     **All Classes** |  |
| SUMMARY: NESTED \| FIELD \| CONSTR \| METHOD | DETAIL: FIELD \| CONSTR \| METHOD |  |


---


## fern.simulation.controller Class AndController

```
java.lang.Object
  fern.simulation.controller.AndController
```

**All Implemented Interfaces:**: SimulationController

---

``` public class AndController extends Object implements SimulationController ```

Implements a `SimulationController` composed of many `SimulationController`.
If one controller tells to go on, the simulation will go on.

**Author:**
:   Florian Erhard

**See Also:**: `OrController`

---

**Constructor Summary**

`AndController(SimulationController... controllers)`


| **Method Summary** | |
| --- | --- |
| `boolean` | `goOn(Simulator sim)`             Returns whether or not to go on with the given simulation |

**Methods inherited from class java.lang.Object**

`clone, equals, finalize, getClass, hashCode, notify, notifyAll, toString, wait, wait, wait`

**Constructor Detail**

### AndController

```
public AndController(SimulationController... controllers)
```


**Method Detail**

### goOn

```
public boolean goOn(Simulator sim)
```

:   **Description copied from interface: `SimulationController`**
:   Returns whether or not to go on with the given simulation

    :   **Specified by:**: `goOn` in interface `SimulationController`
    :   **Parameters:**: `sim` - simulation **Returns:**: whether or not to go on


---


|  |  |  |
| --- | --- | --- |
| **Overview** \| **Package** \| **Class** \| **Use** \| **Tree** \| **Deprecated** \| **Index** \| **Help** | |  |
| PREV CLASS   **NEXT CLASS** | **FRAMES**    **NO FRAMES**     **All Classes** |  |
| SUMMARY: NESTED \| FIELD \| CONSTR \| METHOD | DETAIL: FIELD \| CONSTR \| METHOD |  |


---
